# Local immune response of Canarian Majorera goats infected with *Teladorsagia circumcincta*

**DOI:** 10.1186/s13071-021-05145-y

**Published:** 2022-01-15

**Authors:** Leire Ortega, Jessica Quesada, Antonio Ruiz, María Magnolia Conde-Felipe, Otilia Ferrer, Francisco Rodríguez, José Manuel Molina

**Affiliations:** 1 Parasitology Unit. Department of Animal Pathology, Faculty of Veterinary Medicine, University of Las Palmas de Gran Canaria, Gran Canaria, Spain; 2 Department of Anatomy and Compared Anatomy Pathology, Faculty of Veterinary Medicine, University of Las Palmas de Gran Canaria, Gran Canaria, Spain

**Keywords:** *Teladorsagia circumcincta*, Goat, Abomasal mucosa, Immunoglobulins, Cellular response

## Abstract

**Background:**

Due to increased anthelmintic resistance, alternative methods to drugs are necessary to control gastrointestinal nematodes (GINs). Some of the most promising alternatives are based on the immune response of the host, such as the selection of genetically resistant breeds or the use of vaccines against these parasites. Given the limited information available on the immune response against GINs in goats, this study investigated the local immune response of goat kids of an indigenous Canary Islands breed (Majorera breed) experimentally infected with *Teladorsagia circumcincta*, one of the most pathogenic and prevalent GIN species.

**Methods:**

For this purpose, the relationship between different parasitological (number of mature and immature worms, worm length, and number of intrauterine eggs) and immunological parameters at the local level (related to both the humoral and cellular immune response) was analyzed at early (1 week post-infection [wpi]) and late (8 wpi) stages of infection.

**Results:**

Primary infection of goat kids with *T. circumcincta* infective larvae (L3) generated a complex immune response that could be defined as Th2 type, characterized by increased infiltration in abomasal tissues of several effector cells as well as a progressive presence of specific antibodies against parasitic antigens in the gastric mucus. Cellular responses were evidenced from 1 wpi onward, showing an increase in antigen-presenting cells and various lymphocyte subsets in the gastric mucosa.

**Conclusions:**

The complexity of the host response was evidenced by statistically significant changes in the number of all these subpopulations (MHCII^+^, CD4^+^, CD8^+^, γδ^+^, CD45R^+^, IgA^+^, and IgG^+^), as well as in the evolution of the relative cytokine gene expression. From a functional point of view, negative associations were observed between the number of most of the immune cells (CD4, IgA, IgG, and CD45R cells) and parameters that could be related to the fecundity of worms, a phenomenon that was especially evident when the number of IgG and CD45R cells or the specific IgA levels of the gastric mucus were compared with parasitological parameters such as the female worm length or fecal egg counts at 8 wpi.

**Graphical Abstract:**

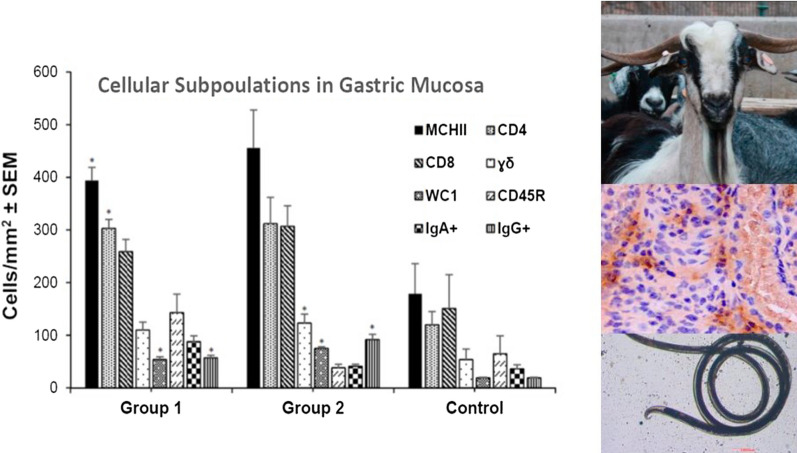

## Background

Gastrointestinal nematodes (GINs) are responsible for important economic losses in livestock farming worldwide, mainly in extensive production systems. Among other effects, these parasites compromise animal welfare and can reduce productivity in terms of milk production, growth rates, carcass quality, and reproductive success. Even in subclinical infections, the presence of these nematodes results in suboptimal use of feed resources and contributes to the development of other infectious diseases [1]. Among the various nematodes that affect the gastrointestinal tract of small ruminants, *Teladorsagia circumcincta* stands out for its wide distribution and pathogenicity [2].

At present, control of these parasitic infections in ruminants relies heavily on treatments with anthelmintic drugs. However, the emergence of parasite resistance to these products [3], as well as consumer concerns about the presence of chemical residues in meat and milk, have stimulated research towards the development of new control strategies [4], as those based on natural products as an alternative to synthetic anthelmintic drugs [5].

Selection of resistant breeds [6] and improvement of the host's immune system by inducing protective responses [7] have also been considered as viable alternatives. Therefore, it would be of great interest to advance the knowledge of the immunological mechanisms generated against ruminant GINs, information that would be very useful for optimizing the development of vaccines against this group of parasites [8].

Though there is an increased interest in goat production as demonstrated by the continuous rise in this livestock population worldwide [9], most recent studies on the immune response to GINs have been conducted in cattle and sheep [10], and much less information is available for goats. In addition, information obtained in cattle or sheep cannot be directly extrapolated to goats [11], as GIN infections are more severe in goats than in other ruminants, there is a delay in the immune response development in goats, making it less effective [12, 13], and finally, different goat breeds have genetic variability in resistance to GIN infections [14, 15]. For these reasons, it is necessary to carry out specific studies to elucidate the immune response generated in goats against this group of nematode parasites.

In goats in particular, *T. circumcincta* constitutes one of the most relevant GIN species due to (i) a high prevalence in many regions of the world [16, 17], (ii) a high pathogenic potential, also in subclinical infections [1, 18], and (iii) a particular tendency to develop resistance to anthelmintics (associated with different factors such as the off-label use of these drugs in goats) [19].

Therefore, the aim of this study is to contribute to the knowledge of the local immunological responses of goats to primary infections with *T. circumcincta* as a preliminary step in uncovering the mechanisms involved in host resistance against this nematode species. In this case, the study focused on the Canary Island Majorera goat, a dairy breed farmed for the production of high-quality cheeses mainly in the eastern Canary Islands, under both intensive and semi-extensive production systems.

## Methods

The *Teladorsagia circumcincta* strain used in this study was originally isolated from a sheep naturally infected with this nematode. It was kindly provided by Dr. Uriarte from the Agrifood Research and Technology Centre of Aragón (CITA, Zaragoza, Spain). This strain was maintained in our laboratory by passage in healthy goats.

Fifteen healthy 3-month-old goat kids (Canarian Majorera breed) from a farm located in the south of Gran Canaria were used in this study. The animals were reared under nematode-free conditions until the beginning of the experiment, at 6 months of age. Experimental animals were randomly allocated to one of the following weight-balanced groups: group 1 (1 week post-infection; *n* = 5), group 2 (8 weeks post-infection; *n* = 5), and group 3 (uninfected control; *n* = 5). Animals from groups 1 and 2 were orally inoculated with 8000 *T. circumcincta* infective third-stage larvae (L3) and slaughtered at 1 (group 1) or 8 (group 2) weeks post-infection (wpi), respectively. Group 3 animals were kept as uninfected controls and were also euthanized at 8 wpi.

Fecal samples were collected three times per week, from day 0 post-infection (pi) until the end of the study (8 wpi) in order to determine fecal egg counts (FEC). Taking into account the prepatent period of *T. circumcincta* infection, coproscopic determinations were only carried out in groups 2 and 3.

At the end of the study, abomasa of all animals were removed to perform parasitological (immature and adult worm counts and length determinations), immunological (specific antibody levels in gastric mucus and antigenic recognition of proteins from *T. circumcincta* adult worms by immunoblot), and histological and immunohistochemical analysis. In addition, samples were collected from gastric mucosa and abomasal lymph nodes to analyze the relative expression of cytokines in both tissues.

### Parasitological analysis

Fecal egg counts (FEC) were determined by the modified McMaster technique [20] using a saturated salt solution (specific gravity 1.20 g/ml at 20 °C) and expressed as number of eggs per gram of feces (EPG). At the end of the experiment, the abomasa of all animals were washed with distilled water, and 200 ml samples were collected and preserved in formalin to determine the number of immature or adult worms (males and females). Thirty immature (group 1) or adult female worms (group 2) from the gastric content of each animal were measured with a calibrated ocular scale. The number of intrauterine eggs per female was also microscopically determined. Finally, immature worm burden in gastric mucosa was established by digestion of mucosal scrapings with pepsin-HCl at 37 °C. Digestion was stopped with formalin and the digestion mix was microscopically examined to calculate the number of immature worms per gram of mucosa, according to sample weight [21].

### Analysis of humoral responses in gastric mucus

Antigen used to analyze the humoral responses was obtained from adult worms collected from the abomasum of monospecific infected donor animals. The worms were homogenized on ice with a homogenizer (Ultra-Turrax T8, IKA^®^ Werke) in a solution of phosphate-buffered saline (PBS) (0.01 M) containing EDTA (1 mM) and phenylmethylsulfonyl fluoride (PMSF) (10 mM) (Sigma-Aldrich, USA). The homogenate was centrifuged at 5000×*g* for 20 min at 4 °C, and the supernatant, containing the soluble somatic antigen, was filtered and its protein concentration determined using a colorimetric method (Pierce BCA Protein Assay, Thermo Fisher Scientific, USA).

Mucus samples used to determine the levels of specific local immunoglobulins were obtained by superficial scraping of the abomasal mucosa. Samples were diluted in a buffer containing proteinase inhibitors (0.1 M sodium phosphate, 0.05 M sodium chloride, 3 mM sodium acid, 1 mM PMSF and 5 mM EDTA; pH 7.2) at a ratio of 2.5 ml buffer/g of mucus. Finally, the samples were centrifuged at 18,000×*g* for 30 min at 4 °C and the supernatant used for the determination of specific antibody levels [22].

#### Enzyme-linked immunosorbent assay (ELISA) test

Optimal test conditions were established based on the results obtained from two pools of positive and negative mucus samples. The final concentrations of somatic antigen used for the determination of specific immunoglobulin G (IgG) or IgA levels in mucus were 3.0 µg/ml or 5.0 µg/ml, respectively. Samples were diluted (1:100 for IgG or 1:25 for IgA) in PBS (0.8% w/v NaCl, 0.02% w/v KCl, 0.144% w/v Na_2_HPO_4_, 0.024% w/v KH_2_PO_4_; pH 7.2), and conjugate (anti-goat IgG-peroxidase [Sigma-Aldrich Inc., USA] or anti-goat IgA-peroxidase [Acris GmbH, Germany]) was diluted in PBS and used at a 1:1000 or 1:5000 dilution, respectively.

A citric acid-phosphate buffer containing 0.04% (w/v) o-phenylenediamine dihydrochloride (OPD) and 0.1% (v/v) H_2_O_2_ was used as substrate. All samples were analyzed in duplicate; the optical density (OD) was determined at a wavelength of 492 nm (Multiskan Ascent 354, Thermo Labsystems, USA) [22].

#### Immunoblot

Somatic antigens of *T. circumcincta* adult worm were fractionated by sodium dodecyl sulphate–polyacrylamide gel electrophoresis (SDS-PAGE) into a 12% (w/v) acrylamide gel under non-reducing conditions. The protein fractions were then electrotransferred onto a 0.22-µM-pore nitrocellulose filter membrane (pure nitrocellulose blotting membrane, BioTrace™ NT, Life Sciences, USA). Then, the nitrocellulose strips were subsequently blocked with a 3% (w/v) solution of bovine serum albumin in PBS. After washing the strips with PBS-Tween 20 (0.2% v/v Tween 20 in PBS pH 7.2), they were subjected to similar steps to those described in the previous section (ELISA test) [23] in which gastric mucus samples were analyzed (diluted 1:1 in PBS). A 1/1000 dilution in PBS of the same conjugates used in ELISA tests were employed. The immunorecognition was evidenced using a staining kit (AEC Staining Kit, Sigma-Aldrich, USA), following the manufacturer's instructions. This kit contained a solution with the chromogen 3-amino-9-ethylcarbazole as substrate, which precipitates on the antigenic fractions detected by peroxidase-conjugated antibodies.

### Histology and immunohistochemistry of abomasal mucosa

Abomasum mucosa tissue samples were cut (4 μm thick) and stained with Giemsa and hematoxylin–eosin to determine the number of eosinophils, globule leukocytes, and mast cells. Cell counts were carried out at a ×500 magnification in 40 randomly selected fields of 0.044 mm^2^, at the upper and lower third of the mucosa. Eosinophils and globule leukocytes were counted in the hematoxylin–eosin-stained sections and mast cells in the Giemsa sections. The results were expressed as the number of cells/mm^2^ [24].

For immunohistochemical analysis, sections (4 μm thick) from the abomasal mucosa were transferred to poly-l-lysine hydrobromide (Sigma-Aldrich Inc., USA) covered slides. Primary monoclonal antibodies against cluster of differentiation 4 (CD4), CD8, CD45R, gamma delta (γδ), major histocompatibility complex class II (MHCII) and workshop cluster 1 (WC1) lymphocytes were diluted at 1:15, 1:15, 1:5, 1:10, 1:20 and 1:5, respectively, in 20% v/v fetal bovine serum in PBS. Polyclonal anti-goat IgG (Vector Labs., USA) and anti-human IgA (Agilent Technologies, USA) sera were used at a dilution in PBS of 1:5000 and 1:500, respectively. All samples were incubated for 90 min at 25 °C. Positive reactions were demonstrated by incubation with biotinylated rabbit anti-mouse immunoglobulins (Agilent Technologies, USA) diluted 1:20 in RPMI medium (Sigma-Aldrich Inc., USA) and a solution of avidin–biotin peroxidase (ABC) complex at a 1:100 dilution in PBS. Finally, slides were reacted with 0.035% (w/v) 3-3′-diaminobenzidine tetrahydrochloride (Sigma-Aldrich) containing 0.01% (v/v) hydrogen peroxide. Counterstaining was performed using Harris' hematoxylin stain; immunoreactive cells were counted in 40 fields located in the upper and lower third of the mucosa [22].

### Determination of relative cytokine gene expression by real-time polymerase chain reaction (RT-PCR)

Abomasal lymph node (approximately 0.5 g) and abomasal mucosa samples (0.5 × 1 × 1 cm) were preserved in TRIzol (TRI Reagent, Sigma-Aldrich, USA) at −80 °C prior to RNA isolation. Total RNA extraction was performed using a double phenolic extraction technique [25]. To eliminate possible contamination with genomic DNA, the samples were treated with DNase (RQ1 RNase-Free DNase, Promega, USA). RNA samples were quantified using a spectrophotometer (NanoDrop 1000 Spectrophotometer, Thermo Fisher Scientific, USA) at 260 nm, and their purity was estimated by determining the OD ratio 260/280 nm, while RNA integrity was determined by means of the RNA 6000 Pico Kit (Agilent Technologies, USA).

Complementary DNA (cDNA) synthesis was performed on a total volume of 20 μl, using 1 μg of total RNA and a reverse transcriptase kit following the manufacturer's instructions (iScript™ cDNA Synthesis Kit, Bio-Rad Laboratories, USA). The resulting cDNA was diluted in 80 μl of DNA-free water (dilution 1:5) before amplification.

RT-PCR was performed using the GoTaq^®^ qPCR Master Mix kit containing Bryt™ Green dye as fluorophore (Promega, USA). The reaction mixture was prepared according to the manufacturer's instructions, however, for most assays, a higher concentration of magnesium chloride (MgCl_2_) was used (Table 1). The amplification process was performed on an iCycler thermal cycler (Bio-Rad, USA) fitted with a MyiQ™ Single-Color Real-Time PCR Detection System. Process monitoring was carried out using the iQ5 Optical System Software Version 2.0 (Bio-Rad, USA). Quantification was performed after 45 denaturation cycles at 94 °C for 15 s, annealing at 61 °C for 20 s and elongation at 72 °C for 15 s.

**Table 1:** Sequence of primers used in qPCR (accession number/reference), size (base pairs [bp]), and melting temperature (Tm, in °C) of amplified products, and MgCl2 concentrations used in each reaction

	Primers 5′ and 3′	Size (bp)	MgCl_2_ (mM)	Tm (°C)	Accession number/reference
Β-act	CCAACCGTGAGAAGATGACCC CCCAGAGTCCATGACAATGCC	122	5	85.0	AF481159
IL-2	GTGAAGTCATTGCTGCTGGA TGTTCAGGTTTTTGCTTGGA	202	3	81.0	Craig et al. (2007) [52]
IL-4	GCTGGTCTGCTTACTGGTATG CGATGTGAGGATGTTCAGC	100	5	80.0	FJ936316
IL-10	GTGGAGCAGGTGAAGAGAGTC TGGGTCGGATTTCAGAGG	198	3	82.0	AF458378
INF-γ	AGATAACCAGGTCATTCAAAGGAG GGCGACAGGTCATTCATCAC	180	3	82.5	U34232
IL-17	TGCTACTGCTTCTGAGTCTGGTGGC TGACCCTCACATGCTGTGGGAAGTT	111	0	83.5	Yan et al. (2011)

The sequence of the primers used for the cytokine gene expression (interleukin 2 [IL-2], IL-4, IL-10, interferon gamma [INF-γ], and IL-17) and β-actin (housekeeping gene) evaluation, the size and melting temperature (Tm) of the amplified products, as well as the concentration of MgCl_2_ used in each reaction, are detailed in Table 1. A relative quantification of gene expression was performed following the method ΔΔCt, comparing cycle threshold (Ct) values obtained from samples of infected and control animals, giving a value of 1 relative unit (RU) to the control group [26]. The data were normalized using the β-actin gene as housekeeping gene.

### Statistical analysis

Data were analyzed statistically using IBM SPSS Statistics software for Windows, version 22.0 (IBM Corp., USA). The differences between the experimental groups in single-day parameters were determined using the nonparametric Mann–Whitney *U* test. Spearman's correlation test was used to analyze the association between different parameters assessed in the study. Probabilities of *P* < 0.05 were considered significant.

## Results

### Parasitological analysis

Infected animals slaughtered at 1 wpi (group 1) showed counts of 1450 ± 228.1 larvae (mean ± standard error of the mean [SEM]), with a mean length of 3.14 ± 0.2 mm. After digestion of the gastric mucosa, 77 ± 33.4 larvae/g mucosa were detected.

In group 2, slaughtered at 8 wpi, the mean (± SEM) of total adult worm counts was 2039 ± 480.7. Female worms (63% of the total number of worms) showed a mean length (± SEM) of 8.32 mm ± 0.6 mm and a mean number of intrauterine eggs (± SEM) of 9.03 ± 2.1 eggs/worm. The number of larvae in the mucosa (mean ± SEM) was 6.6 ± 1.6 larvae/g. Correlation analysis by Spearman's test (*r*) showed a nonsignificant negative association between the number of worms in the gastric content and their length. A statistically significant positive association between length and number of intrauterine eggs was shown (*r*_(3)_ = 0.900, *P* = 0.037).

**Fig. 1:**
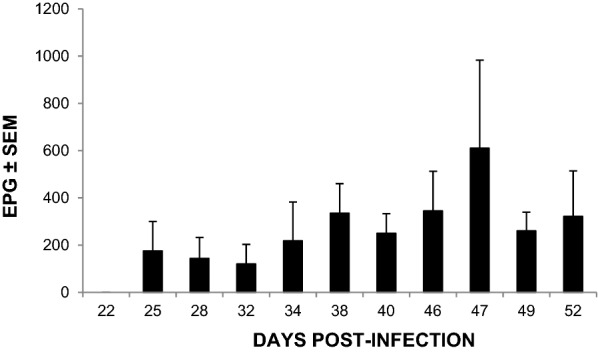
Eggs per gram of feces (EPG) expressed as mean EPG ± SEM in goats orally infected with 8000 *T. circumcincta* L3 (group 2)

Prepatent period of experimental infection in group 2 was 25 days. However, one of the animals showed FEC later on, at day 35 pi. The maximum mean value was reached at 47 dpi (620 EPG) showing a mean FEC of 370 EPG ± 80.5 at the end of the experiment (Fig. 1).

### Analysis of humoral responses in the gastric mucus

#### Specific IgA anti-*T. circumcincta*

**Fig. 2:**
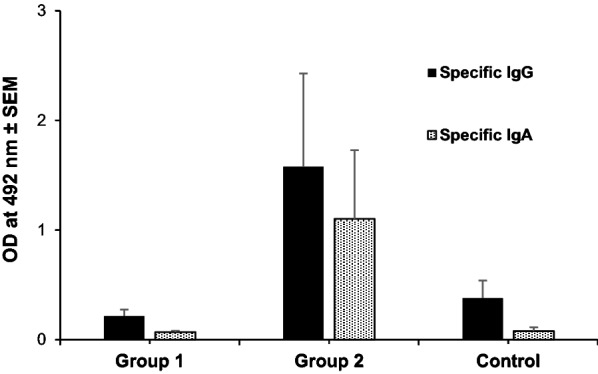
Total IgG and IgA anti-*T. circumcincta* in mucus from groups 1 and 2 (orally infected with 8000 *T. circumcincta* L3 and slaughtered at 1 or 8 wpi, respectively) and the uninfected control group. Results are mean optical density (OD) at 492 nm ± SEM

**Fig. 3:**
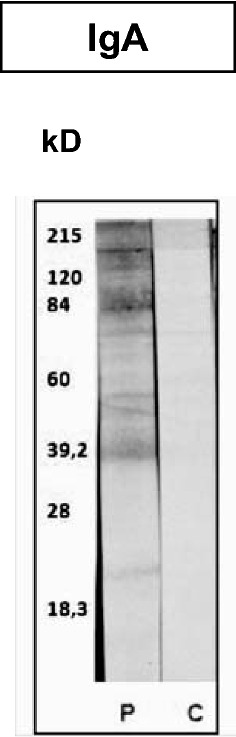
Immunorecognition of mucosal IgA of *T. circumcincta* adult worms somatic antigen in goats from group 2 (orally inoculated with 8000 *T. circumcincta* L3) (**P**) and uninfected control group (**C**) at 8 wpi. *kDa* molecular weight markers in kilodaltons

At the local level, the presence of specific IgA in the gastric mucus was much more evident in infected animals in which worms had reached the adult stage (group 2) compared to group slaughtered at 1 wpi (group 1) and the uninfected control group (Fig. 2). Mucus samples from groups 1 and 3 developed a similar and poor antigenic recognition against somatic proteins of adult worms by immunoblot. In contrast, in animals slaughtered at 8 wpi (group 2), IgA anti-*T. circumcincta* developed a strong reaction against antigenic fractions with molecular weights of approximately 22, 39, 50, 84 and > 120 kilodaltons (kDa) (Fig. 3). In this group 2, the levels of specific antibodies of the IgA isotype present in the gastric mucus were negatively associated with the length of the female worms and the FEC at the end of the study (8 wpi) (*r*_*(3)*_ = −0.975, *P* = 0.005).

#### Specific IgG anti-*T. circumcincta*

As described for IgA response to somatic antigens, the highest specific IgG levels were detected in samples obtained from group 2 (8 wpi), while animals from group 1 and uninfected controls showed a poor specific response (Fig. 2). These levels of mucus-specific IgGs in group 2 were negatively associated with worm length and FEC at slaughter, although no statistical significance could be proven as high variability within the group was found. Despite the relatively high levels of IgG anti-*T. circumcincta* observed by ELISA in samples from group 2, immunoblot could only clearly demonstrate an immunoreaction of IgGs from these samples with a protein fraction with a molecular weight of approximately 39 kDa.

### Histological and immunohistochemical analysis of abomasal mucosa

**Fig. 4:**
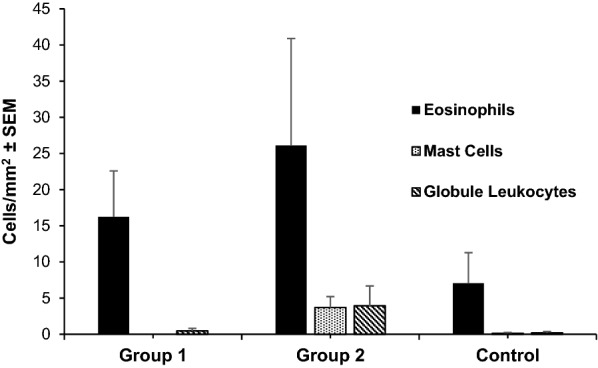
Levels of effector cells (eosinophils, mast cells, and globule leucocyte counts) in the gastric mucosa in goats from groups 1, 2 (orally infected with 8000 *T. circumcincta* L3 and slaughtered at 1 or 8 weeks pi, respectively) and uninfected control group. Results are mean number cells/mm^2^ ± SEM

Infected groups 1 and 2 showed a clearly higher mean number of eosinophils infiltrated in the gastric mucosa than the uninfected control group, although with no statistical significance. The globule leukocytes and mast cells counts were also higher in the gastric mucosal from animals of the infected groups, but similarly these differences were not statistically significant (Fig. 4). In the case of globule leucocytes, a significant negative association was observed between the number of these effector cells and the length of immature worms in the animals from group 1 (1 wpi) (*r*_(3)_ =  −0.894, *P* = 0.041). No statistical associations were observed between the other two effector cell populations (eosinophils and mast cells) and any of the parasitological variables studied.

**Fig. 5:**
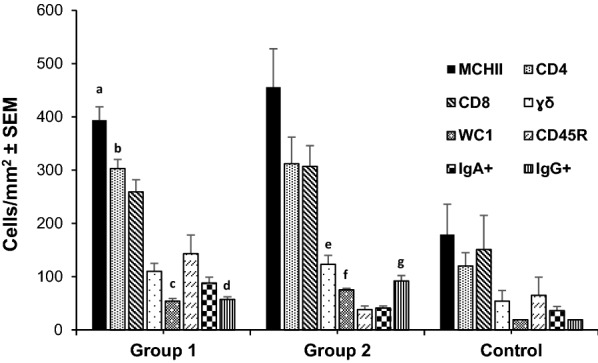
Levels of cellular subpopulations in the gastric mucosa in goats from groups 1 and 2 (orally infected with 8000 *T. circumcincta* L3 and slaughtered at 1 or 8 wpi, respectively) and the uninfected control group. Results are mean number of cells/mm^2^ ± SEM. Abbreviations: Mann–Whitney *U*-test group 1 vs. control: **a** *Z* = −2.193, *P* = 0.028. **b** *Z* = −1.984, *P* = 0.047. **c** *Z* = −2.402, *P* = 0.016. **d** *Z* = −2.601, *P* = 0.09. Mann–Whitney *U*-test group 2 vs. control: **e** *Z* = −2.193, *P* = 0.028. **f** *Z* = −2.402, *P* = 0.016. **g** *Z* = −2.611, *P* = 0.009. MHCII^+^: antigen-presenting cells MHCII. CD4: lymphocyte subset CD4^+^. CD8: lymphocyte subset CD8^+^. γδ: lymphocyte subset γδ^+^. WC1: lymphocyte subset WC1^+^. CD45R: lymphocyte subset CD45R^+^. IgA^+^: anti-IgA immunoreactive cells. IgG^+^: anti-IgG immunoreactive cells

The monoclonal antibodies used in this study showed immunoreactivity with different cell subpopulations of the abomasal mucosa of kids. In both infected groups (groups 1 and 2), all subpopulations of cells studied showed higher mean counts than in the control group. These differences were significant when comparing WC1^+^, CD4^+^, MHCII^+^, and IgG^+^ cells in group 1, and γδ^+^, WC1^+^. and IgG^+^ in group 2 (Fig. 5).

Correlation analysis between parasitological data and immune cell counts in the abomasal wall showed positive associations between IgA^+^ cells and immature worm burden at 1 wpi (group 1). Female worm counts at 8 wpi (group 2) were also positively associated with CD4^+^, γδ^+^ and IgG^+^ cells. On the contrary, negative correlations were detected between CD4^+^, γδ^+^, IgG^+^, IgA^+^ or CD45R^+^ cells and worm length (at both 1 and 8 wpi). At 8 wpi, these subpopulations of lymphocytes (CD4^+^, γδ^+^, IgG^+^, IgA^+^ or CD45R^+^) also showed a negative association with FEC. However, all these negative correlations were not significant except when the relationship between FEC and IgG^+^ cells (*r*_(3)_ = −0.875, *P* = 0.038) or FEC and CDR45R^+^ (*r*_(3)_ = −0.900, *P* = 0.004) cells were analyzed at 8 wpi (group 2).

### Relative cytokine gene expression

**Fig. 6:**
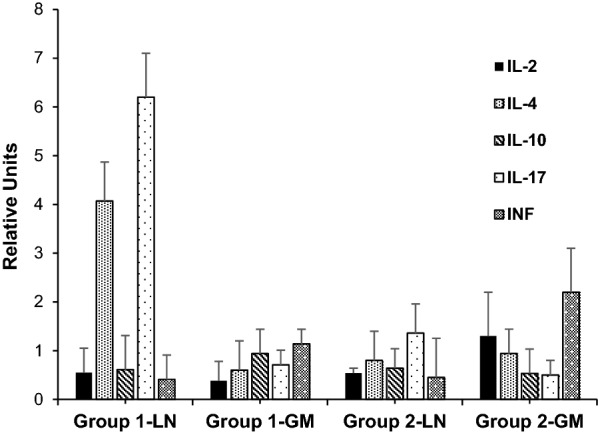
Gene transcription levels of IL-2, IL-4, IL-10, IL-17, and INF-γ in abomasal lymph nodes (LN) and gastric mucosa (GM) from groups 1 and 2 (goats orally infected with 8000 *T. circumcincta* L3 and slaughtered at 1 or 8 wpi, respectively). Results are mean relative units (RU) ± SEM after applying the ΔΔCt method (giving a value of 1 RU to the control group) and using β-actin as housekeeping gene

All experimental groups showed detectable gene transcription levels of IL-2, IL-4, IL-10, IL-17, and INF-γ in both lymph nodes and gastric mucosa (Fig. 6). Although some trends in the relative gene expression between infected and control groups were observed, these differences did not reach statistical significance. In particular, the upregulation of IL-4- and IL-17-gene transcription (fourfold and sixfold, respectively, in comparison with controls) which was detected in the gastric lymph nodes 1 wpi (group 1) was remarkable, whereas this increased gene expression was not detected once the adult worms had developed (group 2). In this latter group, only a slight increase in INF-γ- gene transcription (twofold relative to the control group) was detected in the gastric mucosa.

## Discussion

Despite the homogeneity of the animals included in the different experimental groups in terms of age, sex, breed, weight, origin or feeding, the different parasitological parameters analyzed in the present study showed high variability, as observed in other experimental infections with *T. circumcincta* in Canarian goats [23, 27, 28]. Variability within groups was reflected by high SEM values in some of the parameters assessed, such as larvae and adult worm counts at the end of the experiment, indicating that immunoprotection against the parasite may differ among individuals, as has previously been reported in some ovine breeds infected with *T. circumcincta* [29–31]. This finding has also been described in other goat breeds infected with different GIN species [32], which would indicate that important individual factors are involved in the natural defense mechanisms of goats against GINs.

Similarly, the prepatency period in our study was approximately 25 days, which seems to indicate a slight delay in larval development compared to sheep, which has also been observed in other trials in goats using other isolates of *T. circumcincta* [11], a phenomenon which could be linked to the use of sheep-adapted strains in both cases. The prepatent period reached 35 days in one of the experimentally infected animals, furthering the idea of the presence of a different degree of natural resistance in this animal population that could be manifested by a delay in the endogenous cycle of the parasite.

In sheep, among the mechanism associated with resistance to *T. circumcincta* infections is the reduction in FEC, mainly linked to decreased female fecundity rather than number of parasites [33]. In our study, FEC observed at the end of the experiment (8 wpi, group 2) were negatively associated with the specific humoral immune responses of the two analyzed isotypes (IgG and IgA), pointing to the possible role of the humoral response in the fecundity regulation of *T. circumcincta* in goats. Fecundity was also assessed by the intrauterine egg counts, which showed a logical positive correlation with length of these worms, a feature found in other studies for this parasitic species [30]. Negative correlations also reached statistical significance when analyzing the association between specific IgA levels and female length. This observation could contribute to the idea of the predominant role of the humoral response in the control of *T. circumcincta* fecundity in this ruminant species.

The relevant function of IgA in resistance against *T. circumcincta* has also been observed in sheep infected with this parasite [34–37]. In this ruminant species, it has also been observed that some of these protective responses are associated with an increased antigenic recognition of parasite proteins by IgA immunoglobulins [38]. In the current experiment, the high level of specific IgA in the gastric mucus of infected goats could be related with the higher number of antigenic fractions recognized by immunoblot. With regard to the levels of specific IgG, no association between this isotype and parameters related to resistance were shown. The local humoral response detected at the earliest stages of the infection was scarce. At 1 wpi, this response did not seem to play a relevant protective function, supporting the idea that the animals were not infected with the parasite before the experiment.

The humoral response observed in the gastric mucus corresponds to a Th2 immune response, which is considered a common feature in GIN infections. Th2 response was more evident in the infected group at 8 wpi (group 2). This group showed a significant increase in the level of anti-*T. circumcincta* immunoglobulins (IgA and IgG isotypes) at the local level. This significant enhancement was accompanied by other findings associated with Th2 responses, such as eosinophil infiltration of the mucosa and hyperplasia of mast cells and globular leukocytes [39]. Although tissular eosinophilia has been described as a possible defensive mechanism against *T. circumcincta* [40] or *Haemonchus contortus* [24] in sheep, no significant negative associations between tissular eosinophil counts and parasitological parameters were observed in our study.

The enhancement in the gastric mucosa of other effector cells, such as globule leukocytes and mast cells, has also been associated with natural resistance mechanisms against GINs [41]. Both cell populations increased in the gastric mucosa of *T. circumcincta*-infected goats, reaching higher counts at 8 wpi (group 2), but were also observed in the early stages of infection (group 1, 1 wpi), a finding that could be associated with innate defensive mechanisms [42]. Eosinophils and mast cells also increased rapidly in the abomasal mucosa of goats primo infected with *H. contortus* [43, 44]. However, these authors could not determine an early globule leucocyte hypertrophy as observed in our study. Interestingly, a significant negative association between immature worm length and globule leukocytes at 1 wpi was detected. The potential role of these cells within natural resistance mechanisms in this goat breed should be further evaluated [45].

The present results also showed a rapid recruitment of lymphocyte subpopulations in the abomasal mucosa of goats primarily infected with *T. circumcincta,* similar to that found in goats infected with other gastric nematodes, such as *H. contortus* [46]. This finding was observed from 1 wpi onwards and was characterized by a negative association between some cell subpopulations such as antigen-presenting cells (MHCII^+^) and CD4^+^ lymphocytes and worm burden, a finding that has been linked to the resistance of sheep against GINs [47, 48]. The cellular infiltration, from a functional point of view, also highlights a negative association between CD4^+^, γδ^+^, IgA^+^, IgG^+^, or CD45R^+^ and fecundity (reflected by a reduction in female worm length and FEC) at 8 wpi, as observed when the local humoral immune response was analyzed. The presence of a significant CD8^+^ lymphocyte infiltrate observed here was also previously detected in goats and sheep infected with *H. contortus* from 7 to 10 days pi. However, similarly to what occurred in our study, no relationship could be established with the parasitological data [24, 46]. The infiltration of γδ^+^ lymphocytes and their WC1^+^ subpopulation in the gastric mucosa from the first week of infection was also evident here. These cells constitute an important percentage of lymphocytes in goats, especially in young animals [48], and are considered to have important defensive functions, playing a relevant role in the interrelation between innate and adaptive responses against bacteria, viruses, and protozoan parasites. Nevertheless, there is not much information available on their functionality against nematode parasites in ruminants [50]. As has been observed in goats primarily infected with *H. contortus* [46], this cell population here also displayed an increase in the gastric mucosa from the first wpi, showing a negative association with the length of immature (group 1) and female worms (group 2). This finding has also been observed in Canary sheep resistant to *H. contortus*, a phenomenon associated, as in the current trial, to the local humoral response developed by specific IgA antibodies [24] as discussed above. However, this observation contrasts with results derived from sheep infected with *T. colubriformis*, where no significant defense mechanisms attributed to γδ^+^ cells could be identified [51]. This finding could indicate differential biological functions of this cell type on the basis of parasite species, tissular location, and/or host particularities. Although cytokine expression in lymph nodes and gastric mucosa in infected and control animals was not conclusive, a rapid onset in IL-4 expression was observed, as previously described in sheep [52]. This cytokine could stimulate B-cell proliferation and infiltration of CD4, MHCII, and IgG cells in the gastric mucosa, as observed in our study. Regarding the observed increase in IL-17 expression, although it is a cytokine for which not much information is yet available on its role against ruminant GINs, it appears to have an important regulatory function in the defensive responses necessary to maintain immune tolerance [53].

When the experimental infection progressed, although a humoral response could be detected and some effector cell subpopulations were increased, the gene expression of mediators of the immune response was even less evident than during the first week of infection. Thus, only a slight increase in the relative expression of INF-γ in the gastric mucosa of group 2 compared to the control group was observed. This finding, which has also been reported in the course of *H. contortus* or *Ostertagia ostertagi* infection in goats and calves, respectively, could be contradictory if the inhibitory effect of INF-γ on Th2 responses is taken into account [54]. Such observation has been explained as the beginning of the shift towards an antagonistic Th1 response, which could occur in GIN-susceptible animals after a period of development of Th2 responses, as a result of a mechanism of evasion of the immune response [55–57], or even the expression of a mixed Th1/Th2 response that could be effective against GINs [58].

## Conclusions

In conclusion, the primary infection of goat kids with *T. circumcincta* L3 generates a complex immune response that could be defined as Th2 type, characterized by an increased abomasal infiltration of several effector cells (eosinophils, mast cells, and globule leukocytes) as well as a progressive presence of specific antibodies against parasitic antigens in the gastric mucus. These cellular responses were evidenced from 1 wpi onward, showing an increase in antigen-presenting cells (MHCII^+^) and various lymphocyte subsets (CD4, CD8, γδ, CD45R, IgA, and IgG) in the gastric mucosa. The complexity of the responses developed is evidenced by the statistically significant changes in the numbers of all these subpopulations, as well as in the evolution of the relative cytokine gene expression observed in the gastric mucosa and abomasal lymph nodes at both 1 wpi and 8 wpi. From a functional point of view, negative associations were observed between the number of most of these cells (CD4, IgA, IgG, and CD45R cells) and parameters that could be related to the fecundity (such as the length of female worms or FEC at 8 wpi), a phenomenon that was especially evident when the number of IgG or CD45R cells or the specific IgA levels of the mucus were compared with parasitological parameters such as the length of female worms or FEC at 8 wpi. Although more studies would be necessary, this information should be taken into account to evaluate alternative control strategies against *T. circumcincta* based on the host immune response, such as selection programs for resistant goat breeds or vaccination protocols against this nematode species.

## Abbreviations

GINs: Gastrointestinal nematodes
wpi: Weeks post-infection
L3: Third-stage infective larvae
MHCII: Antigen-presenting cells MHCII^+^
CD4: Lymphocyte subset CD4^+^
CD8: Lymphocyte subset CD8^+^
γδ: Lymphocyte subset γδ^+^
WC1: Lymphocyte subset WC1^+^
CD45R: Lymphocyte subset CD45R^+^
IgA^+^: Anti-IgA immunoreactive cells
IgG^+^: Anti-IgG immunoreactive cells
CITA: Agrifood Research and Technology Centre of Aragón, Spain
FEC: Fecal egg counts
EPG: Eggs per gram of feces
PBS: Phosphate-buffered saline
EDTA: Ethylenediamine tetraacetic acid
PMSF: Phenylmethylsulfonyl fluoride
BCA: Bicinchoninic acid
IgG: Immunoglobulin G
IgA: Immunoglobulin A
OPD: o-Phenylenediamine dihydrochloride
OD: Optical density
SDS-PAGE: Sodium dodecyl sulphate–polyacrylamide gel electrophoresis
ELISA: Enzyme-linked immunosorbent assay
AEC: 3-Amino-9-ethylcarbazole
ABC: Avidin–biotin peroxidase complex
RT-PCR: Real-time polymerase chain reaction
RNA: Ribonucleic acid
DNA: Deoxyribonucleic acid
cDNA: Complementary DNA
IL-2: Interleukin 2
IL-4: Interleukin 4
IL-10: Interleukin 10
IL-17: Interleukin 17
INF-γ: Interferon gamma
Tm: Melting temperature
Ct: Cycle threshold
SEM: Standard error of the mean
RU: Relative units
LN: Abomasal lymph nodes
GM: Gastric mucosa
